# LGAAP: *Leishmaniinae* Genome Assembly and Annotation Pipeline

**DOI:** 10.1128/MRA.00439-21

**Published:** 2021-07-22

**Authors:** Hatim Almutairi, Michael D. Urbaniak, Michelle D. Bates, Narissara Jariyapan, Godwin Kwakye-Nuako, Vanete Thomaz-Soccol, Waleed S. Al-Salem, Rod J. Dillon, Paul A. Bates, Derek Gatherer

**Affiliations:** a Division of Biomedical and Life Sciences, Faculty of Health and Medicine, Lancaster University, Lancaster, United Kingdom; b Ministry of Health, Riyadh, Saudi Arabia; c Department of Parasitology, Faculty of Medicine, Chulalongkorn University, Bangkok, Thailand; d Department of Biomedical Sciences, School of Allied Health Sciences, College of Health and Allied Sciences, University of Cape Coast, Cape Coast, Ghana; e Laboratório de Biologia Molecular, Programa de Pós Graduação em Engenharia de Bioprocessos e Biotecnologia, Universidade Federal do Paraná, Curitiba, Brazil; Indiana University, Bloomington

## Abstract

We present the LGAAP computational pipeline, which was successfully used to assemble six genomes of the parasite subfamily *Leishmaniinae* to chromosome-scale completeness from a combination of long- and short-read sequencing data. LGAAP is open source, and we suggest that it may easily be ported for assembly of any genome of comparable size (∼35 Mb).

## ANNOUNCEMENT

We developed an automated genome assembly and annotation pipeline, successfully applying it to six genomes in the parasite subfamily *Leishmaniinae*, namely, (i) Leishmania martiniquensis (MHOM/TH/2012/LSCM1, LV760), (ii) Leishmania orientalis (MHOM/TH/2014/LSCM4, LV768), (iii) Leishmania enriettii (MCAV/BR/2001/CUR178, LV763), (iv) Leishmania sp. Ghana (MHOM/GH/2012/GH5, LV757), (v) Leishmania sp. Namibia (MPRO/NA/1975/252, LV425), and (vi) Porcisia hertigi (MCOE/PA/1965/C119, LV43). This paper closes the “protocol gap” (1) for this project by making all methods fully available.

**FIG 1:**
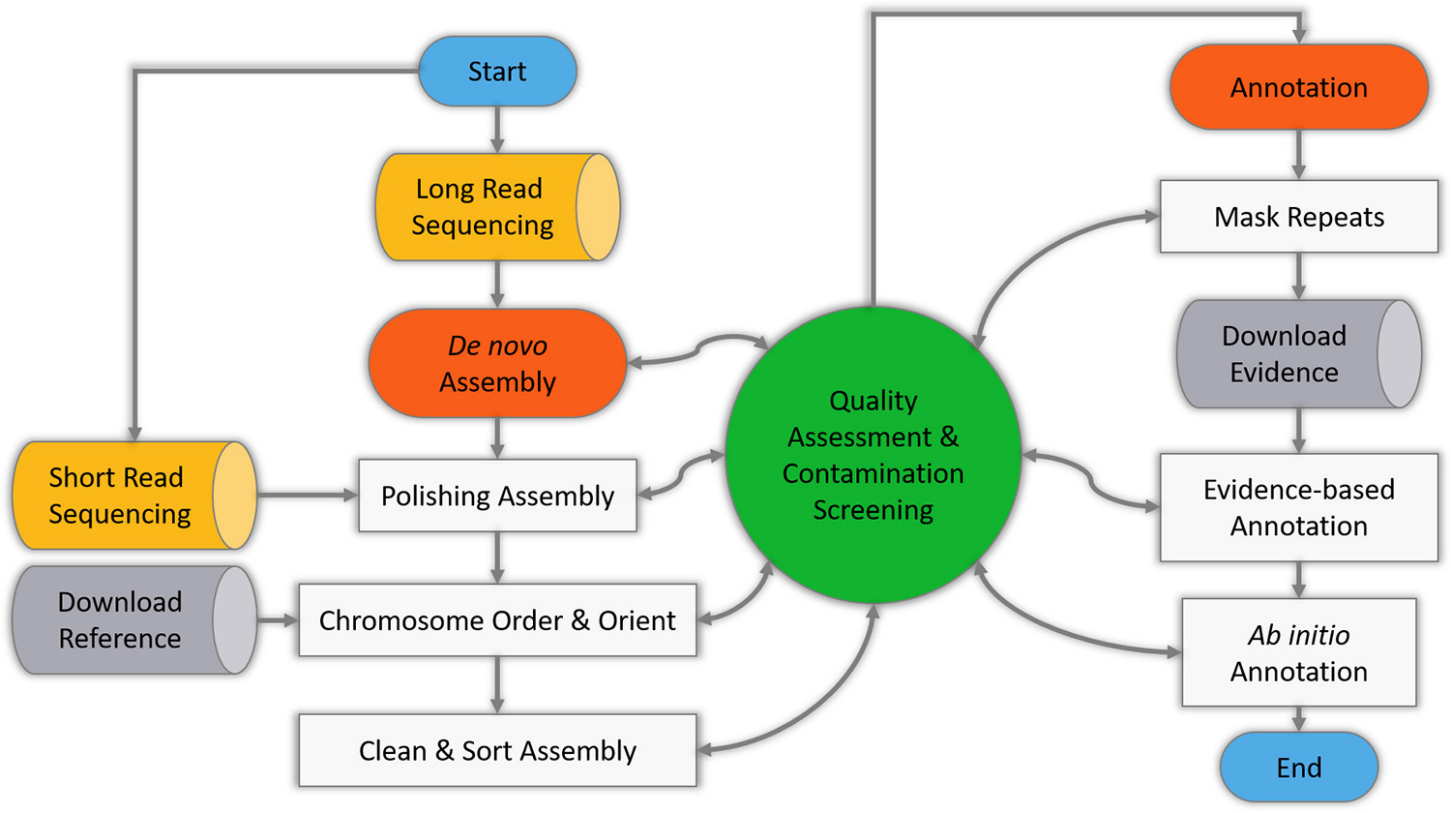
Graphical representation of the LGAAP protocol.

The pipeline was written and executed using the Snakemake (2) workflow management system and consists of a total of 314 computational steps, divided into 21 sequential processes in two main phases (Fig. 1). Genomic DNA was extracted from a previously developed culture system for L. orientalis axenic amastigotes (3) and sequenced using two standard technologies, i.e., short read (Illumina) and long read (Oxford Nanopore Technologies [ONT]).

The first (assembly) phase of the pipeline comprises eight sequential processes, i.e., (i) long-read assembly using Flye (version 2.8.2) (4), (ii) mapping of short reads onto assemblies using Minimap2 (version 2.17) (5), (iii) creation of consensus sequences using SAMtools (version 1.11) (6), (iv) polishing of assemblies using Pilon (version 1.23) (7), (v) revision of consensus sequences using SAMtools, (vi) ordering and orientation of the chromosomes and breakage of any chimeric sequences using RaGOO (version 1.1) (8), (vii) sorting and removal of any duplicated scaffolds or contigs using Funannotate (version 1.5.3) (9), and (viii) generation of a quality report using QUAST (version 5.0.2) (10).

The second (annotation) phase of the pipeline comprises 13 sequential processes, i.e., (i) scanning of assemblies for vector contamination using BLAST+ (version 2.10.1) (11) against UniVec (12), (ii) masking of contaminants using BEDTools (version 2.30) (13), (iii) quality statistics preannotation using AGAT (version 0.6.0) (14), (iv) detection of repeats using RepeatModeler (15) running from Dfam TE Tools Container (version 1.3.1) (16), (v) classification of transposable elements using TEclass (16) running from a docker container (version 2.1.3b) (17), (vi) masking of identified complex repeats using RepeatMasker (version 4.1.2-p1) (18), (vii) downloading of protein and transcript evidence from TriTrypDB (release 47) (19), (viii) evidence-based annotation using MAKER2 (20) running from a docker container (version 2.31.10) (21), (ix) quality checking of annotation using GenomeTools (version 1.2.1) (22) and GAAS (version 1.2.0) (23), (x) *ab initio* annotation using AUGUSTUS (version 3.3.2) (24) within MAKER2, (xi) repeating of the ninth step, (xii) annotation assignments using BLAST+ against UniProt (25) and InterProScan (version 5.22-61.0) (26), and (xiii) finalization of the longest isoforms of each predicted protein using AGAT.

The final product of the analysis pipeline is five files per genome, i.e., the chromosome-scale assembly, proteins, and transcripts in FASTA format and two general feature format (GFF) files, one containing the coordinates of each feature and one with the longest isoforms. Testing on genomes longer than 35 Mb is a future optimization priority. Comparison of the performance of LGAAP with all 50 *Leishmania* genome assemblies in GenBank is shown in Table 1.

**TABLE 1:** Assembly metrics for *Leishmania* genome assemblies deposited in GenBank^*a*^

Organism	NCBI assembly no.	Strain	Sequencing technology(ies)	Assembly method	No. of scaffolds	Total length (bp)	*N*_50_ (bp)
*L. aethiopica*	GCA_003992445	209-622	PacBio RS II	CANU	118	33,648,436	763,733
*L. aethiopica*	GCA_000444285	L147	Illumina	Allpaths-LG	160	31,630,816	1,001,864
*L. amazonensis*	GCA_003992505	210-660	PacBio RS II	CANU	92	33,504,997	850,106
*L. amazonensis*	GCA_000438535	NA	Roche 454, Illumina	Newbler, Velvet, Zorro	2,627	29,029,348	22,901
*L. amazonensis*	GCA_005317125	UA301	Illumina	SMALT	34	32,156,470	NA
*L. arabica*	GCA_000410695	LEM1108	Illumina	AllPaths-LG	168	31,269,090	1,057,807
*L. braziliensis*	GCA_003304975	IOC-L 3564	IonTorrent	SPAdes	1,029	38,003,648	758,103
*L. braziliensis*	GCA_000340355	MHOM/BR/75/M2903	Roche 454	Newbler	744	35,210,150	1,030,512
*L. braziliensis*	GCA_000002845	MHOM/BR/75/M2904	Sanger	NA	138	32,068,771	992,961
*L. braziliensis*	GCA_900537975	MHOM/BR/75/M2904	PacBio, Illumina	NA	35	32,301,632	NA
*L. chagasi*	GCA_014466975	MCER/BR/1981/M6445/Salvaterra	Illumina	SOAPdenovo	36	31,924,566	1,043,794
*L. chagasi*	GCA_014466935	MHOM/HD/2017/M32502/Amapala	Illumina	SOAPdenovo	36	31,924,975	1,043,719
*L. donovani*	GCA_000470725	BHU 1220	Illumina	Bowtie	36	32,414,853	1,024,085
*L. donovani*	GCA_000227135	BPK282A1	Roche 454, Illumina	NA	36	32,444,968	1,024,085
*L. donovani*	GCA_003730175	FDAARGOS_360	PacBio, Illumina	CANU	71	34,011,430	828,097
*L. donovani*	GCA_003730215	FDAARGOS_361	PacBio, Illumina	CANU	56	33,453,722	1,033,854
*L. donovani*	GCA_900635355	HU3	Illumina	NA	36	33,035,865	NA
*L. donovani*	GCA_000283395	Ld 2001	SOLiD^*b*^	Velvet	14,518	27,466,456	3,370
*L. donovani*	GCA_000316305	Ld 39	SOLiD	Velvet	16,323	23,683,296	1,772
*L. donovani*	GCA_003719575	LdCL	PacBio, Illumina	HGAP, Celera Assembler, CANU	36	32,959,864	NA
*L. donovani*	GCA_001989955	MHOM/IN/1983/AG83	Illumina	AllPaths, STLab-assembler	36	32,148,377	1,015,993
*L. donovani*	GCA_001989975	MHOM/IN/1983/AG83	Illumina	AllPaths	36	32,196,393	1,029,368
*L. donovani*	GCA_002243465	Pasteur	PacBio	HGAP	37	33,545,875	1,079,609
*L. enriettii*	GCA_000410755	LEM3045	Illumina	AllPaths-LG	495	30,761,861	868,233
L. enriettii*	GCA_017916305*	MCAV/BR/2001/CUR178, LV763	ONT, Illumina	LGAAP	54	33,318,864	1,075,649
*L. gerbilli*	GCA_000443025	LEM452	Illumina	AllPaths-LG	492	31,398,648	379,527
*L. guyanensis*	GCA_003664525	204-365	PacBio RS II	CANU	123	33,816,023	683,170
*L. infantum*	GCA_003671315	HUUFS14	Illumina	ABySS	2,507	32,578,914	29,848
*L. infantum*	GCA_000002875	JPCM5	Sanger	NA	76	32,122,061	1,043,848
*L. infantum*	GCA_900500625	JPCM5	PacBio, Illumina	NA	36	32,803,248	NA
*L. infantum*	GCA_003020905	TR01	Illumina	Geneious	36	32,009,138	NA
*L. lainsoni*	GCA_003664395	216-34	PacBio RS II	CANU	137	34,152,029	638,860
*L. major*	GCA_000002725	Friedlin	Sanger	NA	36	32,855,089	NA
*L. major*	GCA_000331345	LV39c5	Roche 454	Newbler	849	32,327,517	978,401
*L. major*	GCA_000250755	SD 75.1	Roche 454	Newbler	36	31,242,750	1,022,795
*L. martiniquensis*	GCA_000409445	LEM2494	Illumina	AllPaths-LG	251	30,813,970	873,628
L. martiniquensis*	GCA_017916325*	MHOM/TH/2012/LSCM1, LV760	ONT, Illumina	LGAAP	42	32,413,670	1,046,741
*L. mexicana*	GCA_003992435	215-49	PacBio RS II	CANU	55	32,057,209	825,953
*L. mexicana*	GCA_000234665	MHOM/GT/2001/U1103	Sanger	NA	588	32,108,741	1,044,075
L. orientalis*	GCA_017916335*	MHOM/TH/2014/LSCM4, LV768	ONT, Illumina	LGAAP	98	34,194,276	1,120,138
*L. panamensis*	GCA_000340495	MHOM/COL/81/L13	Illumina	SOAP denovo	952	31,263,945	156,905
*L. panamensis*	GCA_000755165	MHOM/PA/94/PSC-1	Roche 454, Illumina	Newbler, PAGIT	35	30,688,794	1,043,456
*L. peruviana*	GCA_001403695	LEM-1537	NA	NA	37	33,890,200	1,047,715
*L. peruviana*	GCA_001403675	PAB-4377	NA	NA	37	32,907,781	1,015,393
*Leishmania* sp.	GCA_000981925	AIIMS/LM/SS/PKDL/LD-974	Illumina	A5 assembly pipeline	1,100	27,848,322	61,709
*Leishmania* sp. Ghana*	GCA_017918215*	MHOM/GH/2012/GH5, LV757	ONT, Illumina	LGAAP	116	35,953,538	1,100,365
*Leishmania* sp. Namibia*	GCA_017918225*	MPRO/NA/1975/252, LV425	ONT, Illumina	LGAAP	67	34,118,624	1,066,046
*L. tarentolae*	GCA_009731335	Parrot Tar II	PacBio RS II	HGAP	179	35,416,496	663,019
*L. tarentolae*	GCA_009770625	Parrot Tar II	Roche 454	Newbler	7,227	31,556,583	7,432
*L. tropica*	GCA_011316065	ATCC 50129	Illumina	CLC Genomics Workbench	1,928	30,870,161	32,161
*L. tropica*	GCA_014139745	CDC216-162	PacBio RS II, Illumina	Flye	43	32,700,668	1,070,514
*L. tropica*	GCA_000410715	L590	Illumina	AllPaths-LG	448	32,989,014	303,214
*L. tropica*	GCA_003067545	MHOM/LB /2017/IK	Illumina	CLC NGS Cell	9,499	32,139,927	13,854
*L. tropica*	GCA_003352575	MHOM/LB/2015/IK	Illumina	CLC NGS Cell	17,013	32,280,712	7,721
*L. turanica*	GCA_000441995	LEM423	Illumina	AllPaths-LG	336	32,320,007	397,299
Porcisia hertigi*	GCA_017918235*	MCOE/PA/1965/C119, LV43	ONT, Illumina	LGAAP	74	34,958,538	967,170

a Asterisks indicate the six genomes assembled using LGAAP. NA, either not applicable to the technology used or not available from the GenBank record.

b SOLiD, sequencing by oligonucleotide ligation and detection.

### Data availability.

Genomes assembled using this protocol are available in the NCBI Assembly database with the following accession numbers: L. martiniquensis, GCA_017916325.1; L. orientalis, GCA_017916335.1; L. enriettii, GCA_017916305.1; Leishmania sp. Ghana, GCA_017918215.1; Leishmania sp. Namibia, GCA_017918225.1; and Porcisia hertigi, GCA_017918235.1. Raw sequencing data are available with the following NCBI BioProject accession numbers: L. martiniquensis, PRJNA691531; L. orientalis, PRJNA691532; L. enriettii, PRJNA691534; Leishmania sp. Ghana, PRJNA691536; Leishmania sp. Namibia, PRJNA689706; and Porcisia hertigi, PRJNA691541. The workflow is available at GitHub (https://github.com/hatimalmutairi/LGAAP) and Zenodo (https://doi.org/10.5281/zenodo.4663265).
